# Toward Detection of Inert PFAS: Single/Few-CNT Devices for Sensing PFOA

**DOI:** 10.3390/s25247653

**Published:** 2025-12-17

**Authors:** Collins Dormena, Obed Appiah, Taher Ghomian

**Affiliations:** 1 Department of Electrical and Computer Engineering, University of Maine, Orono, ME 04469, USA; collins.dormena@maine.edu (C.D.); obed.appiah@maine.edu (O.A.); 2 Frontier Institute for Research in Sensor Technology, University of Maine, Orono, ME 04469, USA

**Keywords:** carbon, nanotube PFAS, nanosensor, CNT-PFAS interaction

## Abstract

Electron transport in carbon nanotubes (CNTs) is highly sensitive to interactions with their local environment, making them promising candidates for sensing applications. Specifically, this could allow detection of electrochemically and optically inert compounds that typically require complex and expensive analytical techniques. In this study, we examine how single-walled carbon nanotubes (SWCNTs) respond to perfluorooctanoic acid (PFOA), a common per- and polyfluoroalkyl substance (PFAS). To improve sensitivity, we employ a single/few-CNT device setup where a small number of SWCNTs were aligned across nanogaps between gold electrodes with the dielectrophoresis method. This structure addresses the challenges of large CNT networks, such as inter-CNT interactions, drift, and degradation, resulting in improved stability for practical applications. Results showed that device resistance drops as a function of PFOA concentrations. Additionally, positive gate voltage enhances sensitivity by attracting negatively charged PFOA molecules to the SWCNT surface. Specifically, we report that the sensitivity increases by nearly an order of magnitude under a 0.3 V gate bias. Impedance spectroscopy reveals distinct amplitude and phase signatures, enabling selective detection of PFOA among different analytes. Applying gate voltage further enhances sensor selectivity, highlighting the potential of gated SWCNT devices for accurate and selective environmental monitoring. The device demonstrates promising performance as a robust platform for creating single/few-CNT nanosensors for detecting electrochemically and optically inert substances like PFAS molecules.

## 1. Introduction

The use of nanomaterials has rapidly expanded into diverse fields, including electronics, biology, agriculture, optoelectronics, and chemistry [1,2]. Among these materials, CNTs have emerged as a particularly promising platform for sensing applications. This is due to their intrinsic high sensitivity, chemical and mechanical stability, cost-effective fabrication, and structural flexibility [3,4,5,6]. CNTs also have exceptional structural and electronic features, including a high surface-to-volume ratio, atomic-scale thickness, tunable surface chemistry, adjustable bandgap, and excellent electrical conductivity. These properties make them suitable for many sensing mechanisms and easy to integrate into different device architectures [4,7,8,9].

Generally, CNT-based sensors exploit properties such as high surface area, porosity, π–π interactions, and large polarizability to enhance the interaction of CNTs with their surroundings, promoting the adsorption of analyte molecules. The interaction between CNTs and analytes can greatly affect electronic transport properties, depending on factors such as analyte affinity, molecular concentration, and operating temperature [10,11,12,13]. These effects arise from different mechanisms, such as interfacial phonon (RIP) scattering [14,15] and the formation of interface states [16].

**Figure 1:**
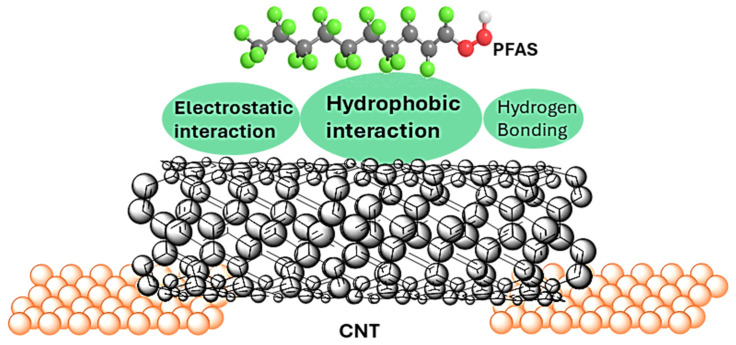
The interaction of PFAS molecules with CNTs in a single/few-CNT device. Electrostatic, hydrophobic, and hydrogen bonding are PFAS-CNT sorption mechanisms. This interaction affects the conduction process in CNTs.

However, per- and polyfluoroalkyl substances (PFAS), including perfluorooctanoic acid (PFOA), remain exceptionally challenging to detect using conventional CNT sensors. Due to their strong C–F bonds, weak polarizability, and absence of π-systems, PFAS molecules exhibit minimal π–π and van der Waals interactions with CNTs [17,18]. Instead, PFAS primarily interact with CNTs through electrostatic forces, hydrogen bonding, and hydrophobic interactions [19,20], as illustrated in Figure 1. Among these, hydrophobic interaction has been reported as the predominant mechanism of PFAS adsorption onto CNTs [20,21]. These weak and non-covalent interactions often yield minimal conductance modulation, leading to low detection sensitivity and poor selectivity compared with more reactive analytes. The excellent performance for PFAS removal from contaminated aqueous media using CNTs [22,23] further shows the effectiveness of this mechanism [17,18,19]. Notably, Pristine CNTs exhibit stronger PFAS adsorption compared to counterparts with oxygen-containing functional groups such as carboxyl and hydroxyl surface groups [19] Additionally, hydrophobic adsorption has been observed to increase with PFAS chain length [17,18,21]. Given these trends, this study focuses on investigating the effect of PFOA, a long-chain PFAS comprising eight carbon atoms and commonly found in environmental samples, on the conductance behavior of pristine SWCNTs in single/few-CNT nanodevices. SWCNTs are chosen because of their environmental sensitivity and exceptional electronic properties, including ballistic transport and long mean free paths, making them a promising platform for next generation sensing technologies.

PFAS molecules are generally electrochemically and optically inert, presenting a significant challenge for detection using conventional techniques. As such, exploring alternative sensing mechanisms is essential. In this context, investigating the influence of PFOA on the electrical conduction of SWCNTs offers a novel strategy for indirect detection based on analyte-induced modulation of transport properties arising from the mentioned interaction mechanisms. Understanding how PFOA adsorption influences the conductance of SWCNTs could lead to selective and efficient detection methods for these environmentally persistent contaminants.

Traditional sensor designs based on a large network of CNTs suffer from a low signal-to-noise ratio and performance drift due to the inter-CNT gap variability and layer degradation. In contrast, low-dimensional devices composed of a small number of SWCNTs demonstrate improved electrical stability and sensitivity due to reduced structural complexity and stronger local interactions [6].

Since the early demonstration of single-walled carbon nanotube field-effect transistor (SWCNT-FET), CNT-based devices have emerged as powerful platforms for chemical and biological detection owing to their high surface-to-volume ratio and tunable electrical properties [24,25]. These foundational studies revealed that even minor charge transfer or dipole interactions at the CNT surface can produce measurable conductance changes, laying the groundwork for later developments in gas, biosensing, and environmental monitoring applications. Building upon these early insights, the present study explores the use of a single/few-CNT configuration toward the detection of PFAS compounds, which remain challenging for conventional sensing techniques.

In this study, we investigate the effect of PFOA adsorption on the conductance behavior of SWCNTs in single/few-SWCNT devices, demonstrating the potential of this approach as a low-cost, simple, and rapid method for PFAS detection. The low dimensionality, combined with gate-field modulation, provides a platform that directly overcomes key limitations of previous CNT-based sensors by amplifying the PFOA-SWCNT interactions and improving device stability through the elimination of network-induced drift.

Efficient and reliable methods for precisely positioning high-quality SWCNTs on a substrate are essential for fabricating low-dimensional single/few-CNT nanosensors. Although various techniques such as Langmuir-Schaffer [26], meniscus drying [27], floating evaporative self-assembly [28], shear-flow [29], growth and transfer [30], and deep coating [31] have been developed for nanotube deposition, all suffer from accurate control over the location of individual CNTs. While chemical vapor deposition (CVD) is a common method for growing CNTs on a substrate, the high-temperature process and challenges in producing small-diameter and high-quality CNTs prevent these methods from fulfilling the requirements of technological applications [32].

**Figure 2:**
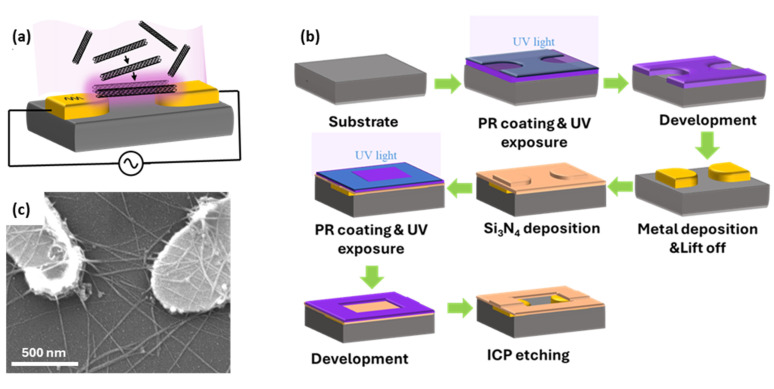
(**a**) Schematic of the DEP process showing a non-uniform electric field generated by nanoelectrodes attracts nanotubes toward the gap between nanoelectrodes. (**b**) Photolithography for the high-throughput fabrication of the chips containing nanoelectrodes suitable for the DEP process. (**c**) The SEM image of a typical device showing a few SWCNTs crossing nanoelectrodes.

Dielectrophoretic (DEP) manipulation is a method that can accurately place CNTs in predefined locations and attach them to electrodes. This method also allows the use of commercially available high-quality, high-purity CNTs to utilize their unique properties. In this work, DEP is used as a post-growth manipulation technique to accurately place SWCNTs between two gold nanoelectrodes while preserving their structure and properties. DEP describes a phenomenon where a force is applied on a dielectric material when it is placed in a non-uniform electric field (see Figure 2a) [2,33]. The dielectrophoretic force is given by:

(1) $$F_{DEP}=\frac{1}{2}\alpha \cdot \nabla (E^{2})$$

where α is the polarizability of the object and E is the electric field. This force moves the particle either along or against the field gradient, depending on the dielectric properties of the target material and suspending media [34]. Typically, this process requires fabricating at least a pair of electrodes sized for the target nanomaterial.

## 2. Experimental Section

The fabricated device consisted of two 50 nm thick gold nanoelectrodes connected to contact pads via gold wires on a sapphire/silicon substrate. SWCNTs were positioned between the gold nanoelectrodes using the DEP method. The following sections describe the fabrication of nanoelectrodes and the process used for SWCNT trapping.

### 2.1. Nanoelectrode Fabrication

Figure 2b shows a schematic overview of the nanoelectrodes and DEP chip fabrication process. Nanoelectrodes were fabricated using a photolithography method to lower costs and increase throughput compared to electron-beam lithography. While electron beam lithography enables the fabrication of thinner electrodes compatible with one-dimensional materials, a custom-built monitoring system is employed in wider nanoelectrodes fabricated with the photolithography technique to prevent excessive accumulation of nanotubes in the trapping area. The fabrication started with spin-coating of a 270 nm thick layer of high-resolution positive photoresist (KL 5302) onto a clean substrate, followed by soft baking to remove residual solvent. The wafer was then exposed to UV light through a patterned mask. After a second soft bake, the exposed regions were developed with MF CD-26 developer. Next, a 5/60 nm thick Cr/Au layer was deposited via e-beam evaporation, with the chromium layer improving gold adhesion to the substrate. The process continued with a lift-off step to remove unwanted metal layers and photoresist. To precisely control the trapping location, an insulating layer should be applied over all chip connections, leaving only the trap area exposed to the analyte solution. For this, a 100 nm thick silicon nitride (Si_3_N_4_) layer was deposited across the entire chip surface using plasma-enhanced chemical vapor deposition (PECVD). To define open areas, standard photolithography was used. A 1-µm-thick layer of positive photoresist (S1813) was spin-coated onto the Si_3_N_4_ layer, followed by soft baking to remove solvents. The wafer was then exposed to UV light through a patterned mask, followed by a second soft bake and development in MF CD-26 to dissolve the exposed regions. The exposed Si_3_N_4_ was etched using ICP etching. Finally, a wet cleaning process removed residual photoresist and organic contaminants, completing the chip fabrication. The DEP chip contained 14 devices with three trap sizes: 400 nm, 600 nm, and 800 nm. The nanoelectrodes connected to the contact pads enabled trapping and characterization.

### 2.2. DEP Manipulation of SWCNTs

The nanotube trapping process began with wet cleaning of the fabricated DEP chip using solvents to remove surface contaminants. For the final surface treatment, the cleaned chip was placed in a Harrick Plasma Cleaner. A prefabricated chamber made of Polydimethylsiloxane (PDMS) was bonded onto the surface of the DEP chip. Then, 10 µL of the diluted SWCNT solution purchased from NanoIntegris Technologies Inc. was deposited onto the device to fully cover the electrode gap. A custom-made circuit was designed to control the trapping process and monitor the number of trapped CNTs by measuring the impedance of the sensor. A sinusoidal trapping signal of 2 Vpp at 34.5 kHz was applied to the nanoelectrode through a probe station, while a simultaneous monitoring signal was set at 1 Hz with an amplitude of 0.1 Vpp. The applied trapping signal generated a non-uniform electric field strong enough to guide SWCNTs toward the electrode gap. Although the width of the electrodes considerably exceeded the diameter of the SWCNTs, the integrated monitoring system prevented excessive accumulation of nanotubes in the trapping area. Impedance monitoring of the device continues in real-time to ensure a controlled deposition of SWCNTs. The DEP process stopped once the desired alignment is achieved. Figure 2c presents a typical device in which a few SWCNTs bridge the nanoelectrodes, demonstrating a successful placement of nanotubes using the DEP method.

### 2.3. Characterization Methods

To investigate the effect of PFOA on the conductance of the fabricated devices, current-voltage (I–V) measurements were performed across a range of PFOA concentrations. The chip temperature was kept at 14 °C inside an environmental enclosure to reduce evaporation of the analyte solution, prevent changes in concentration during measurements, and eliminate the impact of temperature variation. A single device was used for all measurements across different PFOA concentrations. After each measurement, the analyte concentration was increased incrementally by adding a high-concentration stock solution to the existing solution within the PDMS chamber. A saturated PFOA solution was first prepared in deionized (DI) water and subsequently diluted with DI water to generate the stock solution. Electrical resistance measurements were performed using a Keithley 2450 Source Meter. Device responses were evaluated by plotting the normalized resistance as a function of the normalized PFOA concentration. Resistance values are normalized to the resistance measured at the lowest PFOA concentration for that device.

To further characterize the system, impedance spectroscopy was carried out across controlled analyte concentrations using a Digilent Impedance Analyzer with an AC excitation amplitude of 100 mV. Spectra were normalized to the corresponding DI water spectrum collected during the same experimental run. To assess the electrostatic effect, we performed I–V measurements on a silicon-based device under varying analyte concentrations and gate voltages. In this measurement, the DI water spectrum at Vg = 0 V was used as the normalization reference.

## 3. Results and Discussion

**Figure 3:**
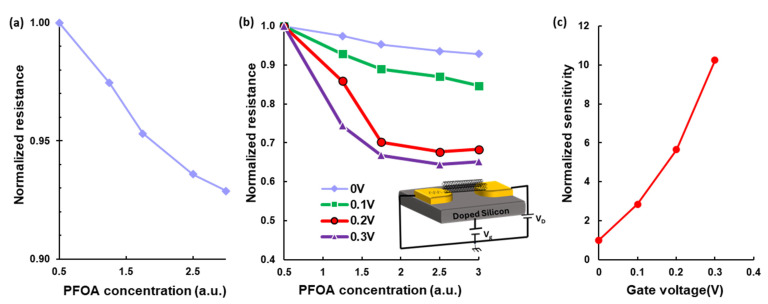
Effect of PFOA concentration and gate voltage on the electrical response of a few-SWCNT device. (**a**) Normalized resistance as a function of normalized PFOA concentration. A 7% reduction in normalized resistance was observed over a sixfold increase in concentration. (**b**) Normalized resistance versus PFOA concentration under varying gate voltages, using a back-gated field-effect structure. Applying positive gate voltages significantly enhanced sensitivity but saturated sensors at high gate voltages. (**c**) Sensor sensitivity in the linear region increases with gate voltage, improving signal-to-noise ratio and enabling detection of lower PFOA concentrations.

Figure 3a shows the normalized resistance as a function of normalized PFOA concentration. For a device with an initial resistance of 1.45 MΩ, a clear 7% decrease in normalized resistance was observed as the normalized PFOA concentration increased by a factor of 6. This indicates that the presence of PFOA molecules alters the electronic properties of the sensor. Overall, the observed trend and linear response support the capability of single/few-SWCNT devices to provide reliable and quantifiable electrical signals for PFOA detection.

Although PFAS adsorption on pristine CNTs primarily occurs through hydrophobic interactions, electrostatic interactions can influence the process [20,21]. PFAS molecules, with a positively charged core and a negatively charged shell, can establish electrostatic interactions with their surroundings. They tend to be attracted to positively charged materials, but since the surface of CNTs is generally negatively charged in water [19], this leads to repulsive electrostatic interactions and adversely affects PFAS adsorption on CNTs. Overall, the high efficiency of CNTs in removing PFAS from contaminated water demonstrates the importance of hydrophobic interactions. While PFAS primarily adsorbs onto pristine CNTs via hydrophobic forces, electrostatic interactions can enhance the overall adsorption process. An interesting approach to controlling the electrostatic interaction between SWCNT and PFAS molecules is the use of a back-gated field-effect structure.

To investigate the electrostatic effect on sensor response, I–V measurements were performed on a device fabricated on a silicon substrate, while varying both analyte concentration and applied gate voltage. In this structure, a doped silicon substrate acts as the gate electrode, and a 300 nm thick silicon nitride layer functions as the insulating dielectric, as illustrated in the inset of Figure 3b. The normalized resistance of the device as a function of PFOA concentration under different gate voltages is shown in Figure 3b. At Vg = 0 V, resistance slightly decreases as PFOA concentration increases. However, applying positive gate voltages improves the device’s sensitivity. At Vg = 0.1 V, the normalized resistance drops by 15% when the PFOA concentration increases by a factor of 6. This response becomes even more prominent at higher gate voltages: a 32% drop is observed at Vg = 0.2 V, and a 35% drop at Vg = 0.3 V under the same concentration change.

These results indicate that higher gate voltage amplifies the sensitivity. However, this increase in sensitivity reduces the linear range of the sensor, as it approaches saturation at lower concentrations with increasing gate bias. Nevertheless, the sensitivity in the linear region rises sharply with gate voltage. Sensitivity in the linear region increases by a factor of 2.8 at Vg = 0.1 V, 5.6 at Vg = 0.2 V, and 10.2 at Vg = 0.3 V, as shown in Figure 3c. This effect results in more significant resistance modulation and improves the signal-to-noise ratio, allowing for more accurate detection of low PFOA concentrations. Figure 3c shows that a small gate voltage of 0.3 V increases the sensitivity by one order of magnitude.

The enhanced PFOA sensitivity observed under positive gate bias can be attributed to the combined effects of electrostatic carrier modulation in SWCNTs and gate-induced adsorption of PFOA molecules to the SWCNT surface. Control experiments in DI water exhibited negligible conductance variation under the gate effect. This confirms that the dominant contribution to the conduction change arises from gate-induced adsorption of PFOA molecules. The increased adsorption under positive gate bias enhances the PFOA-SWCNT interactions, leading to a larger conductance change. Furthermore, the decrease in the sensor’s linear range with increasing gate voltage, as shown in Figure 3b, supports the effect of the adsorption-driven mechanism.

Although previous results demonstrate that the electrical conduction of the device can be modulated by interactions with PFOA, and that applying a gate voltage can boost this interaction, an extra mechanism is needed to specifically distinguish the response to PFAS from other environmental components. This is important because SWCNT conductance remains highly sensitive to various species in the local environment due to their large polarizability.

**Figure 4:**
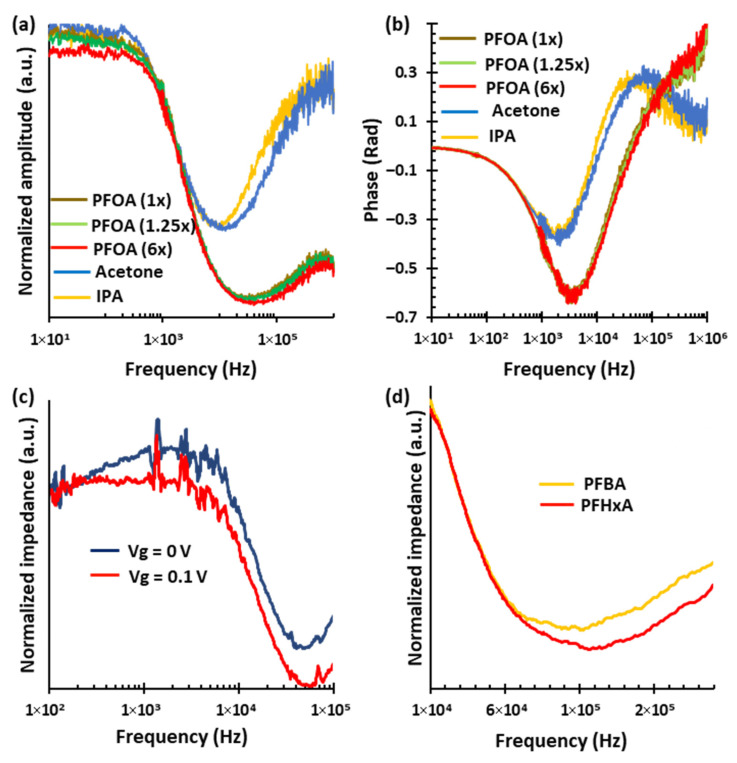
Impedance spectroscopy for selective identification and quantification of PFOA. (**a**) Normalized impedance amplitude spectra for various analytes, including multiple concentrations of PFOA, acetone, and IPA, measured across the frequency range of 10 Hz to 1 MHz. Each analyte exhibits a distinct spectral signature independent of concentration. (**b**) Corresponding impedance phase spectra further differentiate the analytes. (**c**) Normalized impedance amplitude spectra for a fixed PFOA concentration under gate voltages application, over the range of 100 Hz to 1 MHz. The application of a gate voltage lowers the impedance amplitude across most of the spectrum, enhancing spectral resolution. (**d**) Normalized impedance amplitude spectra for PFBA and PFHxA, showing a unique spectral feature independent of concentration.

Figure 4a represents the normalized impedance amplitude spectra for various analytes, including multiple concentrations of PFOA, acetone, and isopropanol (IPA) across the frequency range from 10 Hz to 1 MHz. Each analyte displays a characteristic peak frequency, with acetone peaking at around 11.2 kHz, IPA at 9.3 kHz, and PFOA at 33.8 kHz, regardless of concentration. The spectral profiles confirm that each analyte produces a unique amplitude signature. Within the PFOA group, the amplitude of the spectra decreases systematically as concentration increases. This is particularly distinct in the low- and high-frequency regions outside the peak area. PFOA with a normalized concentration of 6× (red curve) shows the lowest amplitude, followed by PFOA with a normalized concentration of 1.25× (green curve) and PFOA with a normalized concentration of 1× (brown curve). This enables the sensor to differentiate between PFOA concentrations. These distinct spectral features in impedance amplitude spectra support both the selective identification and quantification of PFOA in complex mixtures.

Figure 4b illustrates that the impedance phase spectra provide unique features for distinguishing each analyte and concentration. Notably, the phase responses of PFOA are significantly different from those of organic solvents like acetone and IPA. The PFOA group exhibits a strong negative peak near 3.4 kHz, with a phase minimum around −0.63 rad. In contrast, IPA has shallower negative peaks near 1.8 kHz with phase values close to −0.36 rad, and acetone peaks around 1.9 kHz with a phase of −0.38 rad. Additionally, both IPA and acetone show secondary, distinct peaks at higher frequencies, around 39 kHz and 69 kHz, that have positive phases of 0.27 and 0.28 rad, respectively. The PFOA curves suggest an extra positive phase peak at even higher frequencies, although this feature is outside the current measurement range. Importantly, the PFOA group demonstrates clear separation in phase values above 200 kHz across different concentrations, enabling effective discrimination among PFOA concentration levels. These concentration-dependent variations in phase spectra, along with the consistent peak frequency regardless of concentration, determined by the material’s intrinsic response, highlight the phase spectrum’s usefulness as a complementary, reliable tool for the selective identification and detection of PFOA in complex mixtures.

We also examine the effect of gate voltage on impedance spectroscopy measurements. Figure 4c presents the normalized impedance amplitude spectra for a fixed PFOA concentration under two gate voltages of Vg = 0 V (dark blue curve) and Vg = 0.1 V (red curve), over the frequency range of 100 Hz to 1 MHz. Applying a 0.1 V gate voltage boosts the effect of PFOA by lowering the impedance amplitude across a wide range of the spectrum, thereby improving the visibility and resolution of key spectral features. This reduction is attributed to the mentioned gate-induced attraction of PFOA molecules toward the SWCNT surface, which effectively increases the local analyte concentration and enhances its interaction with the sensor. Such modulation is especially similar to what is observed at low frequencies, where molecular interactions are stronger. Using gate voltage thus allows for clearer and more reliable differentiation of the analyte response, highlighting its potential to significantly improve both the selectivity and sensitivity of the sensor for PFOA detection. Figure 4d presents the normalized impedance-magnitude spectra for Perfluorobutanoic acid (PFBA), and Perfluorohexanoic acid (PFHxA), measured over the 100 Hz to 1 MHz frequency range. Each analyte, independent of concentration, exhibits a distinct spectral signature, enabling clear differentiation among the PFAS species. Specifically, characteristic spectral features appear near 120 kHz for PFHxA and 100 kHz for PFBA. These differences in frequency-dependent impedance behavior provide a promising indication of selective sensing capability among PFAS variants.

The sensing performance of CNT-based devices can be influenced by environmental factors. The variation in ionic strength can modify the electric double layer at the CNT/electrolyte interface, potentially screening the gate field and altering electrostatic interactions with PFAS molecules. In practical environments where multiple PFAS compounds coexist, longer-chain PFAS are expected to absorb strongly onto the CNT surface, which may reduce the detection sensitivity for shorter-chain variants. Future work will therefore involve systematic cross-interference studies under controlled experimental conditions to evaluate the effect of environmental conditions.

While long-term drift and surface fouling remain typical challenges for nanomaterial sensors in liquid media, the design of our platform is expected to substantially mitigate these effects. The single/few CNTs are directly connected to the metal contacts at both ends, eliminating the typical drift and degradation seen in large CNT networks. Surface fouling is expected to be limited due to the chemical robustness of CNTs and the absence of a dense CNT network, which allows adsorbed PFAS or other chemicals to be effectively removed by DI-water rinsing, mild chemical cleaning, or the application of gate voltage. Proper filtering can further remove particles while allowing PFAS molecules to reach the sensing surface. These strategies are expected to support stable device performance over extended operation.

The fabrication process combines standard photolithography for nanoelectrode patterning and DEP alignment for SWCNT placement. The effectiveness of the DEP method has been previously demonstrated by its ability to reliably position a large number of CNT devices within a confined area [33,35]. This achievement highlights the scalability of DEP for parallel assembly of nanoscale devices, enabling high device density and compatibility with advanced CMOS integration.

## 4. Conclusions

This study investigates the effect of PFOA on the conductance behavior of single/few-SWCNT nanodevices. SWCNTs are positioned between two gold nanoelectrodes on a sapphire/silicon substrate. The presence of PFOA molecules affects the charge transport properties. The results demonstrate that increasing PFOA concentration causes a noticeable decrease in the resistance of the device. We further explore the influence of an applied gate voltage on the sensor’s sensitivity. The results show that increasing the gate voltage causes a greater reduction in resistance when exposed to PFOA. We explain that the improved response is because a positive gate voltage generates an electrostatic field that pulls negatively charged PFOA molecules toward the SWCNT surface, leading to a greater decrease in resistance. While gate voltage can boost the sensor’s sensitivity, it also reduces the linear response range and causes the sensor to saturate at higher PFOA concentrations.

Impedance spectroscopy further supports the potential of single/few-SWCNT nanosensor for selective detection of PFOA by revealing analyte-specific spectral features in both amplitude and phase. Besides PFOA, IPA and acetone were tested as representative organic interferents. Each analyte shows distinct responses and characteristic peak positions in both the impedance magnitude and phase angle spectra, regardless of concentration, allowing for selective detection. Additionally, systematic shifts in amplitude and phase are observed with increasing PFOA concentrations, enabling not only identification but also concentration-dependent quantification of PFOA in complex mixtures. Applying a gate voltage also enhances sensitivity and selectivity in impedance measurements by further reducing impedance values and enhancing the distinct features among analytes and their concentrations. Overall, these results highlight the effectiveness of the proposed nanodevices as a promising platform for PFAS sensing applications. This study primarily establishes a proof of concept for PFOA detection using a single/few-CNT device; however, further quantitative evaluation is needed to validate the sensor’s performance.

## Data Availability

The data are available from the corresponding author upon request.
